# A viscoelastic alginate-based hydrogel network coordinated with spermidine for periodontal ligament regeneration

**DOI:** 10.1093/rb/rbad009

**Published:** 2023-02-14

**Authors:** Songbai Zhang, Yuanbo Jia, Jingyi Liu, Fan Feng, Zhao Wei, Min Zhang, Feng Xu

**Affiliations:** State Key Laboratory of Military Stomatology & National Clinical Research Center for Oral Diseases & Shaanxi International Joint Research Center for Oral Diseases, Department of General Dentistry and Emergency, School of Stomatology, Fourth Military Medical University, Xi’an 710032, P.R. China; The Key Laboratory of Biomedical Information Engineering of Ministry of Education, School of Life Science and Technology, Xi’an Jiaotong University, Xi’an 710049, P.R. China; Bioinspired Engineering and Biomechanics Center (BEBC), Xi’an Jiaotong University, Xi’an 710049, P.R. China; The Key Laboratory of Biomedical Information Engineering of Ministry of Education, School of Life Science and Technology, Xi’an Jiaotong University, Xi’an 710049, P.R. China; Bioinspired Engineering and Biomechanics Center (BEBC), Xi’an Jiaotong University, Xi’an 710049, P.R. China; The Key Laboratory of Biomedical Information Engineering of Ministry of Education, School of Life Science and Technology, Xi’an Jiaotong University, Xi’an 710049, P.R. China; Bioinspired Engineering and Biomechanics Center (BEBC), Xi’an Jiaotong University, Xi’an 710049, P.R. China; State Key Laboratory of Military Stomatology & National Clinical Research Center for Oral Diseases & Shaanxi International Joint Research Center for Oral Diseases, Department of General Dentistry and Emergency, School of Stomatology, Fourth Military Medical University, Xi’an 710032, P.R. China; The Key Laboratory of Biomedical Information Engineering of Ministry of Education, School of Life Science and Technology, Xi’an Jiaotong University, Xi’an 710049, P.R. China; Bioinspired Engineering and Biomechanics Center (BEBC), Xi’an Jiaotong University, Xi’an 710049, P.R. China; The Key Laboratory of Biomedical Information Engineering of Ministry of Education, School of Life Science and Technology, Xi’an Jiaotong University, Xi’an 710049, P.R. China; Bioinspired Engineering and Biomechanics Center (BEBC), Xi’an Jiaotong University, Xi’an 710049, P.R. China; State Key Laboratory of Military Stomatology & National Clinical Research Center for Oral Diseases & Shaanxi International Joint Research Center for Oral Diseases, Department of General Dentistry and Emergency, School of Stomatology, Fourth Military Medical University, Xi’an 710032, P.R. China; The Key Laboratory of Biomedical Information Engineering of Ministry of Education, School of Life Science and Technology, Xi’an Jiaotong University, Xi’an 710049, P.R. China; Bioinspired Engineering and Biomechanics Center (BEBC), Xi’an Jiaotong University, Xi’an 710049, P.R. China

**Keywords:** periodontal regeneration, viscoelastic hydrogel, mechano-biochemically synergistic treatment, mechanical microenvironment

## Abstract

Periodontitis can cause irreversible defects in the periodontal ligament (PDL), the regeneration of which is the major obstacle to the clinical treatment of periodontitis. Implanting hydrogel for releasing anti-inflammatory drugs is a promising treatment to promote PDL regeneration. However, existing hydrogel systems fail to mimic the typical viscoelastic feature of native periodontium, which may have been shown as an important role in tissue regeneration. Meanwhile, the synergistic benefits of mechanical cues and biochemical agents for PDL regeneration remain elusive. In this study, we developed a bi-crosslinking viscoelastic hydrogel (Alg-PBA/Spd) by integrating phenylboronic acid-modified alginate with anti-inflammatory agent (spermidine) through borate ester and B–N coordination bonds, where spermidine will be released with the degradation of the hydrogel. Alg-PBA/Spd hydrogel is biocompatible, injectable and can quickly adapt to complex periodontal structures due to the dynamic crosslinking. We demonstrated in rat models that the viscoelastic Alg-PBA/Spd hydrogel significantly promotes the deposition of periodontal collagen and accelerates the repair of periodontal damage. Our results suggest that the viscoelastic Alg-PBA/Spd hydrogel would be a promising mechano-biochemically synergistic treatment for periodontal regeneration.

## Graphical Abstract

**Figure:**
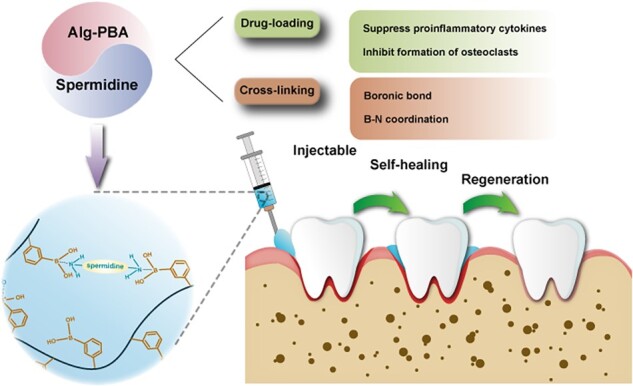

## Introduction

Periodontitis is an inflammatory disease caused by infection of periodontal tissue [1], with the typical feature of irreversible destruction of periodontal ligament (PDL) and alveolar bone [2]. Traditional periodontitis therapies [3] (e.g. periodontal scaling treatment [4], periodontal flap treatment [5], inducing osteogenesis [6]) aim to slow disease progression by eliminating the source of infection and reducing inflammation, but fail to achieve the regeneration of PDL [7, 8]. PDL is a structure composed of mainly collagen fibers, with an amorphous matrix [9, 10]. Such a compositional basis determines the typical viscoelasticity of PDL [11], which can structurally modulate the response of the tooth–PDL–bone complex pair to dynamic loading [12], in turn mediating bone remodeling through dynamic changes of fibers and extracellular matrix [13]. Although PDL regeneration is critical to periodontitis treatment, the complex inflammatory microenvironment of periodontitis [14] and the complex structural [15] and mechanical properties [16, 17] of the periodontium set a huge obstacle to PDL regeneration [18]. Therefore, it is of great importance to develop more effective treatments.

In clinics, various drugs (e.g. tetracycline, chlorhexidine and metronidazole) have been used in the treatment of periodontitis, which can achieve anti-inflammatory function through antibacterial activities [19]. In recent years, traditional anti-inflammatory drugs (e.g. acetylsalicylic acid) are also loaded in implanted repair materials to treat periodontitis [20]. However, the above drugs can only treat a certain pathogenic factor of periodontitis [21]. Therefore, there is still an unmet need to find a multi-functional drug for the treatment of periodontitis. Spermidine is an emerging anti-inflammation agent that inhibits the expression of interleukin 1β (IL-1β) [22] and exerts anti-inflammatory effects [23], which can keep cellular functions and help maintain cellular homeostasis [24]. Spermidine also has many regenerative benefits such as immunomodulation [25], antioxidant functions [26] and inhibition of the formation of osteoclasts [27], which has not been explored yet in PDL regeneration. Besides, spermidine is usually delivered orally [28] or directly encapsulated in hydrogel [29], which results in too rapid drug release to cover the treatment period of periodontitis (∼7 days) [30].

Hydrogels are considered to be a promising regenerative material [31], with the capabilities to provide controlled drug release and mechanical support to tissues [32, 33]. Existing hydrogel-based strategies for PDL regeneration are limited to the delivery of biochemical factors (e.g. drugs and cells) [34], with limited functional recovery of PDL [35]. Accumulating evidence shows that the mechanical properties of hydrogels (e.g. stiffness, viscoelasticity) play important roles in tissue regeneration [36]. In view of the critical role of PDL viscoelasticity in maintaining periodontal function [37], we hypothesize that integrating spermidine therapy with viscoelastic hydrogel may be a promising approach for PDL treatment. However, the mechanical benefits of viscoelastic hydrogels for PDL regeneration and the mechano-biochemically synergetic effect remain elusive.

Many approaches have been developed to construct viscoelastic hydrogels [33], including host–guest interaction, ionic cross-linking and dynamic covalent bonding [38–40]. Among them, borate ester dynamic interaction-based viscoelastic hydrogels have attracted much attention because of their good injectability and viscoelasticity compatible with biological tissues [41]. The structural basis of the borate ester bond is the dynamic ester bond formed by the boronic acid and the *cis*-diol [42, 43], which was widely used in the regenerative medicine and drug delivery [44–46]. Particularly, since primary and secondary amines in spermidine can form a B–N coordination bond with phenylboronic acid [47]. The B–N coordination bond that formed between the secondary amine and the B atom can improve the hydrolytic stability of the entire system [48]. The introduction of spermidine into the borate hydrogel may integrate mechanical viscoelasticity and controlled release of spermidine.

Herein, we report a bi-crosslinking viscoelastic hydrogel (Alg-PBA/Spd) by integrating phenylboronic acid-modified alginate (Alg-PBA) with spermidine (Spd). Alg-PBA can self-gel by binding to *cis*-diols on the alginate backbone, which provides an ideal viscoelastic hydrogel template to study the effect of hydrogel viscoelasticity on PDL regeneration. Spermidine is introduced to the hydrogel network through the B–N coordination bond which exerts anti-inflammatory effects after release from the network with the degradation of the hydrogel. We demonstrated the synergistically therapeutic potential of viscoelastic Alg-PBA/Spd hydrogel in a rat PDL injury model.

## Materials and methods

### Materials

Alginate (Alg, Mw = 20–50 kDa) was purchased from Macklin (Shanghai, China). 3-Aminophenylboronic acid (PBA), N-(3-dimethylaminopropyl)-N′-ethyl carbodiimide hydrochloride (EDC), N-hydroxy succinimide (NHS), MES monohydrate (MES), Methacrylic anhydride (MA) and deuterium oxide were purchased from Aladdin (Shanghai, China). α-MEM, fetal bovine serum (FBS) and Penicillin–Streptomycin (PS) were from Gibco/Thermo Fisher Scientific (USA). Spermidine (Spd), phosphate-buffered saline (PBS), cell counting kit-8 (CCK-8) and Calcein-AM/PI Live-Dead Cell Staining Kit were purchased from Solarbio (Beijing, China). Six-week-old male rats were purchased from the Laboratory Animal Center of the Fourth Military Medical University.

### Synthesis of Alg-PBA

Alg-PBA was synthesized through the esterification of Alg with PBA [42]. Briefly, 1 g Alg was first dissolved in 0.1 M MES buffer to form a 1% (w/v) solution. Then, 500 mg PBA dissolved in 5 ml methyl sulfoxide was added to the Alg solution followed by adding 0.5 g EDC and 0.7 g NHS. The pH of the mixed solution was kept to 4.5–5 for 24 h using 1M NaOH at 25°C (Supplementary Fig. S1A). When the reaction finished, the solution was centrifuged at 7500 rpm/min for 0.5 h to remove unreacted PBA. Finally, the supernatant was dialyzed (MWCO 3500) against deionized water for 5 days and lyophilized. The successful modification of alginate was confirmed by ^1^H NMR spectroscopy (400 MHz JEOL). The degree of modification of phenylboronic acid on alginic acid is calculated by using the molar ratio of the phenylboronic acid unit to the alginic acid group, that is, the ratio of the integral value of the phenylboronic acid unit to the integral value of the alginic acid group.

### Preparation and characterization of Alg-PBA/Spd hydrogel

To construct the hydrogel, solutions of Alg-PBA (4%, 3%, 2% w/v in PBS) and Spd (1% w/v in PBS) were mixed by the ruhr lock at a volume ratio of 20:1 in a 1-ml injection syringe at room temperature to obtain hydrogel with different concentrations of Alg-PBA. At the same time, hydrogel with different final concentrations of Spd (0.05%, 0.15%, 0.25%, w/v) were obtained by changing the concentration ratio of Alg-PBA (4%, w/v) to Spd (1%/3%/5%, w/v). The microstructure of hydrogel was observed by scanning electron microscope (SEM) (Hitachi SU3500).

### Rheological tests of hydrogel

Rheological tests were carried out using a rotational rheometer (Anton Paar MCR 302) at 37°C. After the hydrogel was uniformly mixed in the syringe, 0.2 ml was injected on the plate of the rheometer. The surrounding hydrogel was removed after lowering the rotor height to a distance of 1 mm from the plate. The stiffness of different Alg-PBA concentration hydrogel (Alg-PBA:Spd 20:1 w/v) was investigated by testing the storage (G′) and loss (G″) moduli at a range of 10–0.1 rad/s and 1% strain amplitude over time by frequency sweep. The loss tangent of different Spd concentration hydrogel was measured by the same method as stiffness with constant Alg-PBA concentration (4% w/v). The stress relaxation was assessed by relaxation tests at different constant strains (1%, 5% and 10%) for 300 s. The self-healing properties were tested by storge (G′) and loss (G″) moduli alternating strain cycles of the 5% and 500% strains. The self-healing properties were evaluated by shear-thinning testing with shear rate varying in the range of 0.01–5 Hz.

### Characterization of injectability, self-healing, tensile properties, remodeling and degradability properties of hydrogel

The injectability of hydrogel was characterized by squeezing the hydrogel out through a 12# needle. The self-healing properties of the hydrogel were characterized by cutting-healing experiments. For the swelling and degradation tests, 1 ml of 4% Alg-PBA/Spd hydrogel and a 5-ml centrifuge tube were weighed, respectively. Then the hydrogel and 1 ml of PBS were added to the 5 ml centrifuge tube, and PBS was drained completely every 4 h before weighing. The PBS was carefully aspirated with a pipette, and the remaining mass was weighed; when the mass no longer increased, it was weighed every 24 h until it was completely degraded.

### Drug release characterization of hydrogels

We prepared Alg-PBA/Spd hydrogels and Alg/Ca^2+^ hydrogels containing the same concentration of Spd. For Alg/Ca^2+^/Spd hydrogels, spermidine is directly mixed in the Alg solution and Ca^2+^ is added to form Alg/Ca^2+^ hydrogel. We added 1 ml PBS and 5 mM H_2_O_2_ to 100 µl Alg-PBA/Spd hydrogel and Alg/Ca^2+^/Spd hydrogels and the hydrogels were soaked for 6 days. We collected 500 µl supernate every day, and then added the same amount of PBS or H_2_O_2_. We used high performance liquid chromatography to detect the concentration of spermidine in the supernate and calculated the cumulative release of spermidine. Column: C18 column (4.6 mm × 250 mm, 5 µm), liquid phase: Phase A is ultrapure water, Phase B is acetonitrile, gradient elution (the elution procedure is: 0 min, 40% water, 60% acetonitrile; 5 min, 20% water, 80% acetonitrile; 14 min, 5% water, 95% acetonitrile; 18 min, 40% water, 60% acetonitrile). The detection wavelength is 254 nm, and the column temperature is 30°C.

### Degradation test of the hydrogel *in vivo*

*In vivo* fluorescence images of cy7-labeled hydrogels were taken by an animal imaging system (AniView600, Guangzhou Biolight Biotechnology, China). Alg-PBA was replaced by Alg-PBA with cy7-labeled to prepare a Cy7-Alg-PBA hydrogel. Cy7-amine (Duofluor Inc, China) was grafted with Alg-PBA to synthesize Cy7-Alg-PBA. In short, 200 mg of Alg-PBA was dissolved in 20 ml water, then 10 mg Cy7-amine was added to the Alg-PBA solution with 140 mg of EDC and 100 mg of NHS. One molar of NaOH was used to maintain the pH value of the solution from 4.5 to 5.5 for 24 h. After the reaction was over, it was purified by dialysis with deionized water for 3 days, and then freeze-dried. 0.1 ml Cy7-Alg-PBA/Spd hydrogel was injected under the skin of rats for 9 days to test the degradation of hydrogel *in vivo*.

### Cell culture and biocompatibility of hydrogel

Four percent of Alg-PBA/1% Spd hydrogel was dissolved in the medium and the PDL fibroblast cells (PDLFs) were seeded on 96-well plates at the same cell density for 24 h. From the next day, the PDLFs were cultured for 3 days with different concentrations of hydrogel solutions (0.025–10%). All culture medium was added with 10% FBS and 1% PS at 37°C in 5% CO_2_. The biocompatibility of the hydrogel was characterized by cell death staining and cell proliferation assays. The effect of materials on cell proliferation at 1, 3, 5 days was detected by CCK-8 assay. Dead and live cells were stained using Calcein-AM/PI live/dead cell double staining kit. To further characterize the biocompatibility of the Alg-PBA/Spd hydrogel, PDLFs were seeded in a 6-well plate. After 24 h, 100 µl of Alg-PBA/Spd hydrogel and Alg/Ca^2+^ hydrogel were added to the medium separately. 4%Alg-PBA/Spd hydrogel was evenly spread on the bottom of the 6-well plate to form a hydrogel substrate (thickness: 1 mm). The PDLFs were also seeded in the hydrogel and cultured in 3D for 1 and 3 days. The cells were stained with Calcein-AM/PI dye to detect the state of the cells. Images were taken using a confocal microscope (Olympus FV 3000), and the cell viability was calculated as (number of living cells/number total cells) ×100%.

### Anti-inflammatory properties of the hydrogel

PDLFs were seeded in six-well plates and cultured for 24 h; 10 µg/ml lipopolysaccharide, 10 µg/ml concanavalin (final concentration) and different concentrations of spermidine solutions (final concentration: 0/1/10 µM) were added into the six-well plates with periodontal membrane fibroblasts adherence. After 24 h, we collected the cell culture solution. We used enzyme-linked immunosorbent assays to detect IL-1β and IL-6 in the supernatant.

### Synthesis and characterization of Alg-MA-PBA/Spd hydrogels

One gram of Alg was dissolved into a 1% solution and the solution was pre-cooled to 4°C. 1.2 ml MA (Aladdin, China) was slowly added to the 1% Alg solution at unlight and low temperature. The pH of the reaction was maintained between 8 and 9 for 8 h, which was adjusted by 1M NaOH. Alg-MA was obtained after 3 days of dialysis and lyophilized. Alg-MA-PBA was synthesized by the amidation reaction of Alg-MA and 3-PBA (the same method as the synthesis of Alg-PBA by Alg and 3-PBA) (Supplementary Fig. S1B). Alg-MA-PBA/Spd hydrogel was obtained by mixing with 10 mg/ml Spd in a volume ratio of 20:1, and a photoinitiator of 50 mg/ml LAP (Sigma-Aldrich, USA) was added in a volume ratio of 100:1. The hydrogel was cross-linked under 490 nm blue light for 30 s. At 0.01–10 Hz and 1% strain, the storage modulus and loss modulus of the Alg-MA-PBA/Spd hydrogel were measured by frequency sweep, and the stiffness was compared at a frequency of 1 Hz. The stress relaxation of Alg-MA-PBA/Spd hydrogel and Alg-PBA/Spd hydrogel was measured within 200 s under 1% strain, and the half stress relaxation time was compared.

### Measurement of viscoelasticity properties of PDL *in vivo*

Fresh pig maxillary bones were from the slaughterhouse. The maxillary bone was cut by an electric motor saw to obtain the tooth–PDL–bone complex. The complex was trimmed to ensure a flat shape. The stress relaxation of the pig periodontal membrane was measured by a moving-magnetic biomaterial testing system (BOSE3200, USA) (the compression strain is 10%). The stress relaxation data of pig periodontal membrane were compared with the stress relaxation level of the hydrogel.

### Establishment of the animal model

The rat PDL models were established according to the literature [49]. In brief, we separated the maxillary first molar gingiva with a probe in SD rats (male, 300–350 g) and incised the gingiva along the mesial direction to fully expose the root. Then we used a probe to penetrate into the PDL space to destroy the buccal, lingual, and mesial PDLs. Four percent of Alg-PBA/1% Spd hydrogel was injected into the PDL space to fill the periodontal defect, and the gingiva was sutured. Rats were randomly divided into the following groups: Blank (no treatment), Alg-PBA (viscoelastic hydrogel), Alg-MA-PBA/Spd (elastic drug-load hydrogel), Alg-PBA/Spd (viscoelastic drug-load hydrogel). We euthanized the rats at different time points and harvested periodontal tissue to assess healing. All animal procedures performed in this study were reviewed and approved by the Animal Experimental Ethical Inspection of Fourth Military Medical University (2019-035) and were performed in accordance with the guidelines of the International Association for the Study of Pain.

### Histological evaluation

Rats were sacrificed at 1, 2 and 3 weeks, and the maxilla including the first molars and surrounding soft tissues were completely removed. After being fixed in 4% paraformaldehyde for 3 days, it was transferred to EDTA decalcification solution (neutral) for decalcification. The samples were placed in a shaker at 37°C, the decalcification solution was changed every day, and decalcified for 2 weeks. The decalcified samples were sectioned longitudinally along the long axis of the teeth. H&E staining and Masson staining were used to evaluate the effect of hydrogel on PDL repair, and the arrangement of fibers and collagen volume fraction were observed or calculated.

### Statistical analysis

Porosity, viable cell number, inflammatory area and collagen volume were counted using ImageJ (NIH, Bethesda, MD, USA). Significance analysis was performed using GraphPad Prism, using one-way ANOVA and two-way ANOVA and *t*-test, and the significance level was determined at *P* < 0.05 (*).

## Results and discussion

### Synthesis of phenylboronic acid modified sodium alginate and preparation of Alg-PBA/spd hydrogel

**Figure 1.**
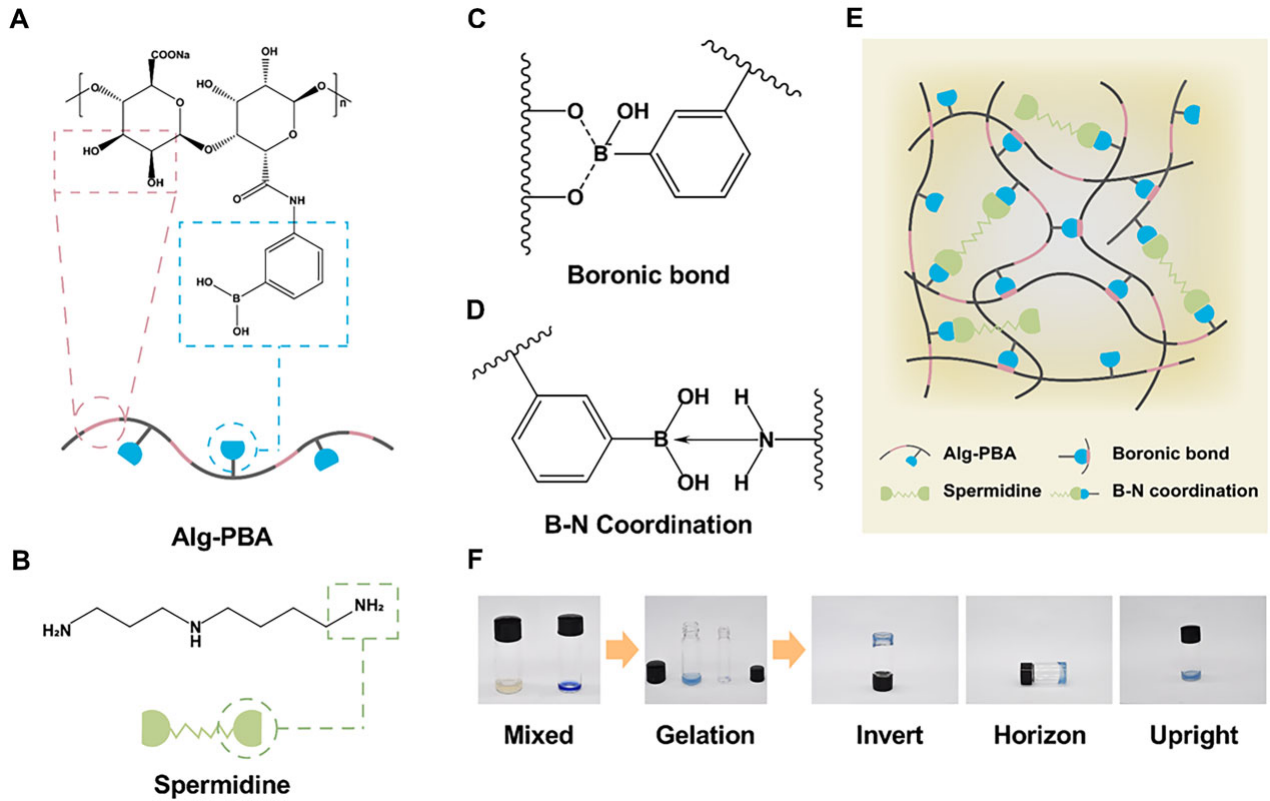
Schematic illustration of Alg-PBA/spd hydrogel. The chemical structure of (**A**) Alg-PBA, (**B**) Spermidine, (**C**) boronic bond and (**D**) B–N coordination. (**E**) Scheme of the preparation process of forming Alginate-PBA/spermidine hydrogel. (**F**) Optical photographs of the Alginate-PBA/spermidine mixture solution and the formed Alg-PBA/spd hydrogel.

To synthesize a dynamic double-crosslinked network hydrogel, we prepared phenylboronic acid modified sodium alginate (Fig. 1A) and Spd (Fig. 1B). The successful grafting of PBA on Alginate was verified by nuclear magnetic resonance hydrogen spectroscopy (NMR) (Supplementary Fig. S1C). By comparing the characteristic peak of the phenyl signal of 7.6 ppm and the reference peak of the alginate skeleton of 4.97 ppm, we calculated that the degree of modification is 21.6%. The hydrogel network is crosslinked by dynamic boronic esters between boronic acid and *cis*-diol in the alginate backbone and further crosslinked by dynamic B–N coordination bonds between boronic acid and amino groups of Spd (Fig. 1C and E). To verify the gelation of hydrogel, we mixed 4% Alg-PBA solution and 1% Spd solution in a ratio of 20:1. Due to the rapid cross-linking and dissociation of the borate bond and the B–N coordination bond, the precursors gel immediately after mixing and the hydrogel does not flow when placed horizontally or upside down (Fig. 1F).

### Characterization of mechanical properties and structure of Alg-PBA/spd hydrogel

**Figure 2.**
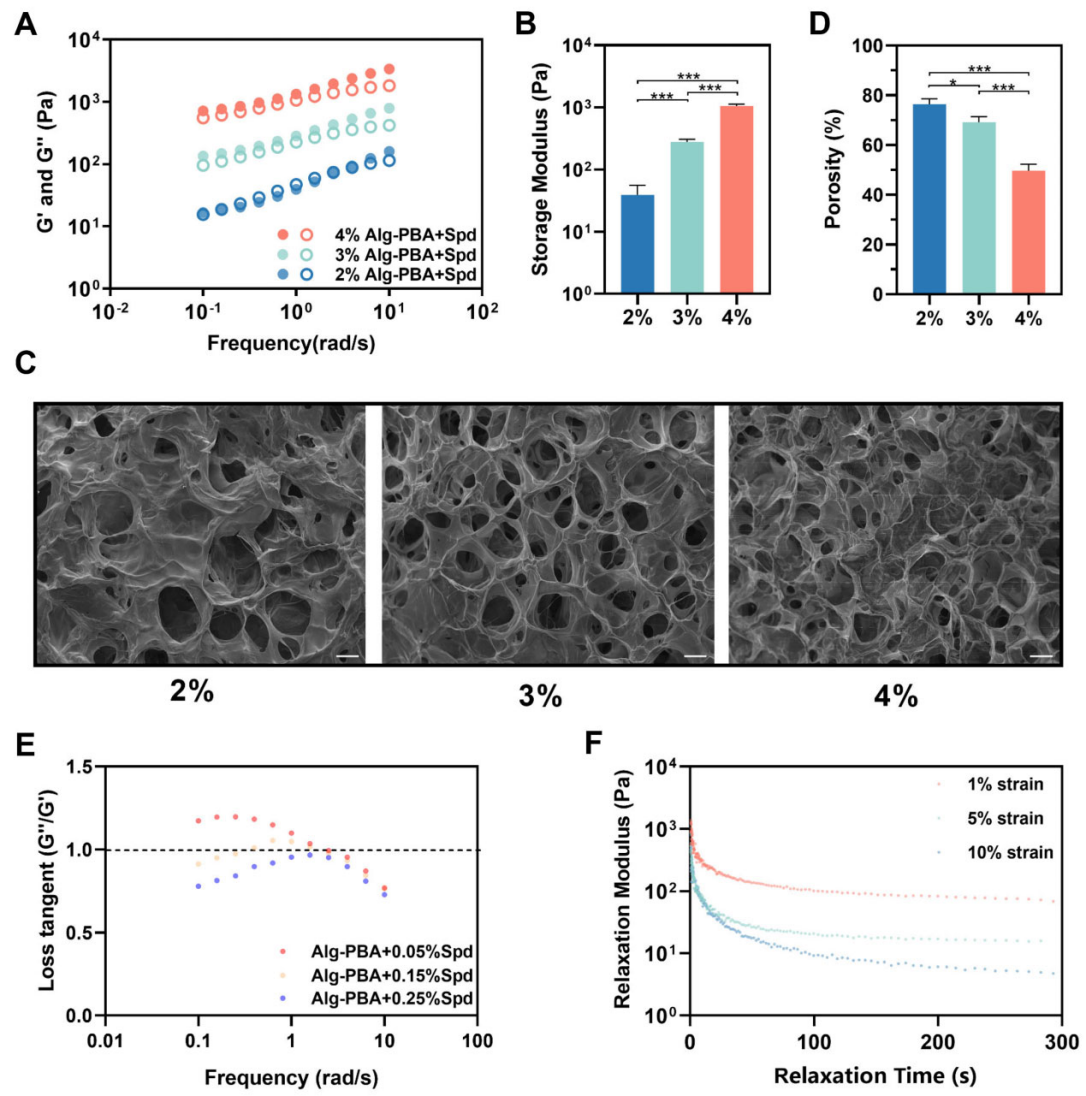
Mechanical properties and structure of Alg-PBA/spd hydrogel. (**A**) Variations of storage modulus and loss modulus (G′ and G″) of Alg-PBA/spd hydrogel with different Alg-PBA concentrations versus angular frequency (0.1–10 rad/s) and 1% strain. (**B**) Storage modulus of Alg-PBA/spd hydrogel with different Alg-PBA concentrations at 1 rad/s frequency and 1% strain. Values are exhibited as mean ± SD. ***P < 0.001. (**C**) Representative SEM images of the Alg-PBA/spd hydrogel. Scale bar = 50 μm. (**D**) Porosity of Alg-PBA/spd hydrogel with different Alg-PBA concentrations. Values are exhibited as mean ± SD. *P < 0.05, ***P < 0.001. (**E**) Loss tangent of the Alg-PBA/spd hydrogel with different spermidine concentrations versus angular frequency (0.1–10 rad/s) and 1% strain. (**F**) Stress relaxation curves of Alg-PBA/spd hydrogel at different strains.

To evaluate the effect of Alg-PBA concentration on the storage modulus (G′) and loss modulus (G″) of Alg-PBA/Spd hydrogel, we mixed different concentrations (2%, 3%, 4%) of Alg-PBA with 1% Spd solution at a ratio of 20:1. We observed that both G′ and G″ increase with the increase of Alg-PBA concentration, but decrease with decreasing angular frequency (Fig. 2A). This is because Alg-PBA/Spd hydrogel composed of dynamic bonds is viscoelastic and there are no frequency bins where the modulus is stable. Therefore, to assess the stiffness of hydrogel with different concentrations, we chose the storage modulus at an angular frequency of 1 rad/s as a reference, with the storage modulus of hydrogel with different concentrations being significantly different (Fig. 2B). To compare the effects of different concentrations of Alg-PBA on the hydrogel porosity, we characterized the microstructure of the hydrogel using SEM and quantified the porosity. We observed that all concentrations of hydrogel exhibit irregular porous microstructures with pore sizes greater than 200 μm and porosity between 50% and 80% (Fig. 2C and D). The porous structure of these hydrogel ensure the transportation of nutrients and oxygen, which is conducive to cell penetration and proliferation, thereby promoting the formation of new tissues. To maximize the drug loading of the hydrogel and improve the stiffness of the hydrogel, we chose 4% Alg-PBA/Spd as the material concentration for the subsequent experiments.

To prove the interaction of spermidine and Alg-PBA, we changed the concentration ratio of 4% Alg-PBA and 1%/3%/5% Spd to check the gel point (loss tangent = 1) of the hydrogel when the loss tangent varies with the angular frequency. We observed that different ratios cause the curves to have different trends, proving the intermolecular interaction between Spd and Alg-PBA (Fig. 2E). According to the results, the gel point of the hydrogel was changed with the different concentration of Spd. In addition, there was a significant difference in the loss modulus of hydrogels with different concentrations at low shear frequency, demonstrating that the concentration of Spd changed the dynamic crosslinking network of the hydrogel. Therefore, these results indicated the formation of the B–N coordination bond between Spd and Alg-PBA.

To characterize the viscoelastic properties of the Alg-PBA/Spd hydrogel, we tested the stress relaxation of the hydrogel under different strains (Fig. 2F). The hydrogel showed a short stress relaxation time, with complete stress relaxation within 100 s under different strains (1%, 5%, 10%). These results indicated that Alg-PBA/Spd hydrogel possessed good viscoelasticity and might be effective in cushioning periodontal stress through rapid relaxation.

### Characterization of injectability, self-healing, tensile properties, remodeling and degradability of Alg-PBA/spd hydrogel

**Figure 3.**
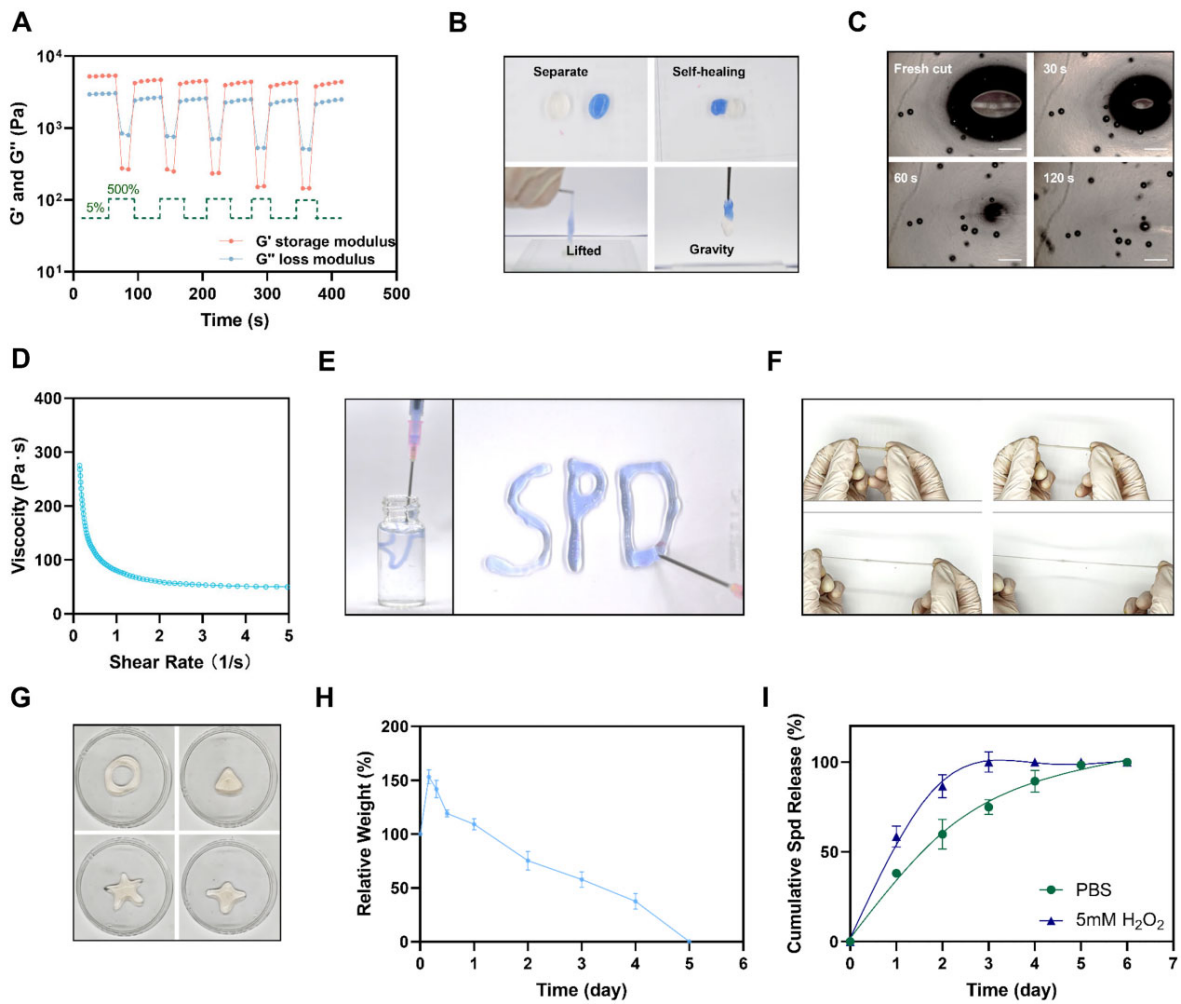
Injectability, self-healing, tensile properties, remodeling, degradability and drug release of Alg-PBA/spd hydrogel. (**A**) Self-healing capacity of Alg-PBA/spd hydrogel by testing the G′ and G″ at alternating strain cycles of 5% and 500%. (**B**) Two pieces of cracked Alg-PBA/spd hydrogels are in contact with each other and the healed hydrogel can support its own weight. (**C**) Monitoring of the self-healing process of a scratch made on an Alg-PBA/spd hydrogel film by optical microscopy. Scale bar = 500 μm. (**D**) Viscosity of Alg-PBA/spd hydrogel with the shear rate from 0.1 to 5 1/s. (**E**) Photographs of the continuous injection of Alg-PBA/spd hydrogel through the 12# needle into any custom-designed shape. (**F**) Photographs of Alg-PBA/spd hydrogel with good tensile properties. (**G**) Photographs of Alg-PBA/spd hydrogel that can be remolded into various shapes. (**H**) Swelling and degradation curves of Alg-PBA/spd hydrogel in PBS. (**I**) The curve of cumulative spd release in PBS and 5 mM H_2_O_2_.

To characterize the self-healing properties of hydrogel, we performed the alternating cyclic strain measurements, and macro- and micro-hydrogel segmentation-self-healing experiments. We observed that Alg-PBA/Spd hydrogel exhibits yielding behavior at the high strain level of 500% (G″ > G′), but the mechanical properties recover rapidly at the low strain level of 5% (G′ > G″) (Fig. 3A). This indicated that the self-healing process can also be repeated many times without compromising the mechanics of the hydrogel. From a macroscopic point of view, we touched two separate hydrogels of different colors and observed that the two hydrogels quickly fuse into a whole, which can be easily lifted resisting its own weight (Fig. 3B). We further incised a piece of hydrogel from the middle and observed that the incision decreases rapidly with complete self-healing within ∼120 s (Fig. 3C). These results demonstrated the self-healing ability and stability of the double dynamic cross-linked network of boronate bond and B–N coordination bond. The self-healing properties of Alg-PBA/Spd hydrogel can greatly improve their application scenarios. When the hydrogel was filled in the defect area of the periodontal tissue, the external force may cause the hydrogel to rupture while the rapid self-healing ability will help to improve the adaptation of the hydrogel to the complex defect patterns.

Considering the narrow and complex spatial structure of periodontal defects, injection is a very important modality for delivering drugs to periodontal tissues. To assess the injectability of the Alg-PBA/Spd hydrogel, we carried out a frequency–viscosity test. The viscosity of the hydrogel decreased significantly with increasing shear rate (Fig. 3D). Besides, the hydrogel can be injected continuously without interruption using a 12# needle into any shape (Fig. 3E). These results suggested that dynamic B–N coordination bonds and borate ester bonds can endow hydrogels with good shear-thinning properties.

To characterize the tensile properties and remodeling of the hydrogel, we stretched the Alg-PBA/Spd hydrogel and observed that the hydrogel can resist a wide range of tensile strain (Fig. 3F) and reshape into different shapes (Fig. 3G). These results indicated that the hydrogel can be adapted to irregularly shaped periodontal defects to achieve perfect filling of the defects, and can remain shape stable under the extrusion of surrounding soft tissues.

To test the degradability of the Alg-PBA/Spd hydrogel, we soaked the hydrogel in PBS solution. We observed that the hydrogel rapidly swells to the maximum volume within 4 h and begins to degrade after 4 h until completely degraded after 5 days (Fig. 3H), which indicated that Alg-PBA/Spd hydrogel can be degraded due to the existence of dual dynamic cross-linking. Since the spermidine was crosslinked with a dynamic bond in the hydrogel, the dynamic bond would be destroyed during the degradation of the hydrogel, providing the synchronous and slow release of spermidine.

To evaluate the spermidine release of Alg-PBA/Spd hydrogel under normal conditions and in the presence of ROS, we tested the drug release of Alg-PBA/Spd hydrogel in PBS and H_2_O_2_ (Fig. 3I). We observed that spermidine is released ∼60% in the first 2 days and ∼80% in the fourth day under normal conditions *in vitro*, indicating the slow and uniform drug release. Compared with normal conditions, H_2_O_2_ can significantly accelerate the release of spermidine, with Spermidine completely released on Day 3. At the same time, we also observed the complete degradation of the hydrogel on Day 3. Our results showed that in the presence of reactive oxygen species in tissues (such as inflammation), Alg-PBA/Spd drugs can accelerate the release and act on inflammatory tissues, thereby performing anti-inflammatory functions faster.

### Comparison of hydrogels between borate bond and ionic bond

Compared with conventional metal ion (Ca^2+^) hydrogels, Alg-PBA hydrogels have advantages in several ways. Conventional metal ions (Ca^2+^) induced gelation through the formation of ionic bonds between Ca^2+^ and alginate can achieve rapid gelation, but the process is physical interactions, which may make hydrogel heterogeneous. The improved calcium ion penetration gelation method will greatly extend the gelation time. As a result, boronate bond can perform fast and achieve well-formed hydrogel.

To compare the mechanical properties of Alg-PBA/Spd with traditional Alg/Ca^2+^ hydrogels, we prepared 4% Alg-PBA/Spd hydrogels and 4% Alg/Ca^2+^ hydrogels (4% Alg was soaked with 0.1M calcium chloride for 30 min). We used frequency sweep to characterize the stiffness of Alg-PBA/Spd hydrogel and Alg/Ca^2+^ hydrogel (Supplementary Fig. S2A). The results showed that the average stiffness of Alg-PBA/Spd hydrogel is lower than that of Alg/Ca^2+^ hydrogel at 1 Hz (Supplementary Fig. S2B). The Alg-PBA/Spd hydrogel has a faster stress relaxation compared with the Alg/Ca^2+^ hydrogel under 10% strain (Supplementary Fig. S2C and D). The ionic bond crosslinking formed between calcium ions and alginate is relatively stable, so it has higher stiffness and worse dynamics. On the contrary, borate bonds have better dynamics, and the hydrogel network is easily re-crosslinked after breaking. So, the stiffness of the hydrogel is lower, and at the same time it has an extremely fast stress relaxation rate.

To compare the *in vitro* biocompatibility of Alg-PBA/Spd hydrogel and Alg/Ca^2+^ hydrogel, we detected the live/dead quantity of the cells on Days 1 and 3 by Calcein-AM/PI dye. We observed that both Alg-PBA/Spd hydrogel and Alg/Ca^2+^ hydrogel have good biocompatibility (Supplementary Fig. S2E).

The spermidine and calcium ions in Alg-PBA/Spd hydrogels and Alg/Ca2 hydrogels are distributed in the body. Spermidine in the human body is mainly derived from food and microorganisms in the intestine, and Ca2 is an important component of the human body. So both Spd and Ca^+^^+^^2+^ in hydrogels have good biocompatibility. In addition, alginate and its derivatives are macromolecular polysaccharides that are widely distributed in nature, so the hydrogels containing alginate also have good biocompatibility.

To compare the drug release of Alg-PBA/Spd hydrogels and Alg/Ca^2+^ hydrogels, we characterized the spermidine release of Alg-PBA/Spd hydrogels and Alg/Ca^2+^ hydrogels loaded with spermidine (Supplementary Fig. S2F). We observed that Alg/Ca^2+^ releases spermidine faster, with more than 80% released within 2 days. In comparison, the spermidine release of Alg-PBA/Spd hydrogel is significantly slower than that of Alg/Ca^2+^. Alg-PBA can form a B–N covalent crosslinking with Spd. With the slow degradation of the Alg-PBA/Spd hydrogel, the B–N coordination bond is broken and the spermidine is slowly released. Alg/Ca^2+^ and Spd are physically mixed to achieve drug delivery, so spermidine is directly released.

### Characterization of the biocompatibility and anti-inflammation

**Figure 4.**
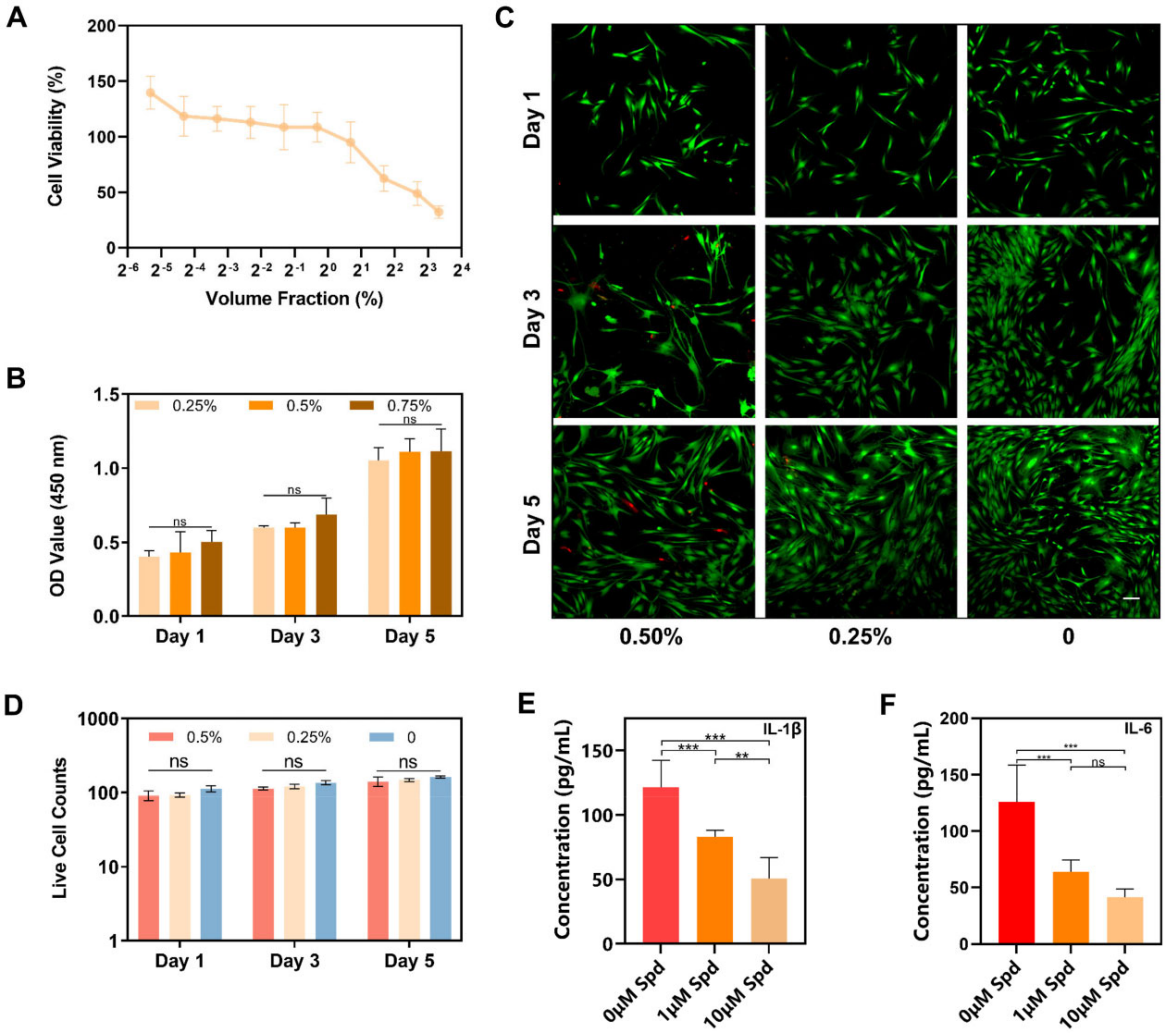
Biocompatibility and anti-inflammation of Alg-PBA/spd hydrogel. (**A**) Cytotoxicity of Alg-PBA/Spd through CCK-8 assay of PDLFs viability after co-culture with different hydrogels mass fraction at specific time points (Day 3). (**B**) CCK-8 assay of PDLFs proliferation after co-culture with different hydrogel mass fraction for Days 1, 3, 5. PDLFs, periodontal ligament fibroblast cells; CCK-8, cell counting kit-8. (**C**) Live/dead staining of PDLFs at specific time points (Days 1, 3, 5) in 1 week of co-culture. Calcein AM staining (green) shows the high viability of cells. Scale bar =100 μm; 0.50%, 0.25%, 0, the mass fraction of Alg-PBA/spd hydrogel. (**D**) Live cell counts of the live/dead staining of PDLFs in (C). (**E**) ELISA analysis of IL-1β secretion in different spd concentrations. (**F**) ELISA analysis of IL-6 secretion in different spd concentrations. Data are shown as mean ± SD and compared using one-way ANOVA followed by Bonferroni’s post hoc test. ns, *, ** and *** indicate *P* > 0.05, *P* < 0.05, *P* < 0.01 and *P* < 0.001, respectively.

To assess the cytotoxicity of the hydrogel, we characterized the cytotoxicity of 4% Alg-PBA/Spd hydrogel for the PDLFs using CCK-8 assay. We observed that when the hydrogel solution concentration is lower than 1% (volume fraction, v/v), the cell viability remained stable (cell viability >100%), while cell viability decreased rapidly with increasing concentrations higher than 1% (Fig. 4A). According to this result, we selected the concentration of hydrogel solution (lower than 1%) to further detect the level of cell proliferation. PDLFs maintain proliferation under different concentrations of hydrogel solutions for 5 days (Fig. 4B).

To further verify the biocompatibility of the hydrogel, we also performed Live/Dead staining experiments. We observed that PDLFs in the experimental groups (0.5% and 0.25%) and the control group kept mostly live (green) with normal spindle-like morphology. After 5 days of culture, the proliferation process was not affected (Fig. 4C). No significant difference was observed among the 0.5%, 0.25% and 0% group at the same time point (Fig. 4D), which remained consistent with the results of the CCK-8. We further evaluated the viability when PDLFs were cultured on Alg-PBA/Spd hydrogel (2D) and in Alg-PBA/Spd hydrogel (3D) for 1 and 3 days (Supplementary Fig. S3A). The cell viability in 2D culture is over 90%, and the viability in 3D is over 75%. The above results all confirmed good biocompatibility of the Alg-PBA/Spd hydrogel.

To evaluate the anti-inflammatory ability of spermidine *in vitro*, we used different concentrations of spermidine to treat PDLFs in the inflammatory state and detected the IL-1β and IL-6 content in the cell culture solution using enzyme-linked immunosorbent experiments (Fig. 4E and F). When the spermidine concentration is 1 and 10 µM, the IL-1β concentration is 82.9 and 50.5 pg/ml, respectively. When the spermidine concentration is 1 and 10 µM, the concentration of IL-6 was 63.9 and 41.6 pg/ml, respectively. Spermidine can significantly reduce the production of IL-1β and IL-6. This result proves that spermidine has good anti-inflammatory ability, which may be an important reason why spermidine can promote periodontal tissue regeneration.

### The degradation of the hydrogel *in vivo* and mucoadhesive property

To test the degradation of the hydrogel *in vivo*, we implanted Cy7-Alg-PBA/Spd hydrogel under the skin of rats, and evaluated the degradation of the hydrogel through quantitative fluorescence intensity for up to 9 days (Supplementary Fig. S4A). The results showed that the hydrogel remained about 40% after 5 days of implantation and ∼20% after 9 days of implantation (Supplementary Fig. S4B). This suggested that the hydrogel in the body can cover the rapid regeneration stage of PDL before degradation completely.

To evaluate the adhesion formation after PBA modification, we designed the related adhesion experiments. We pasted two pieces of fresh pork on the slide and evenly applied Alg-PBA/Spd hydrogel on the surface. We observed that the Alg-PBA/Spd hydrogel pastes two pieces of pork into a whole. When we separated the two pieces of pork, we observed the filamentous hydrogel produced by adhesion between the pork tissues (Supplementary Fig. S5A). The alginate hydrogel modified by PBA can form a *cis*-diol structure, which is similar to mussel adhesion, providing good adhesion properties for alginate. At the same time, the positively charged amino groups in spermidine can be adsorbed on the tissue surface by electrostatic action. The primary amino group can covalently bind to the carboxyl group on the tissue surface, which may enhance the adhesion of the hydrogel.

### Synthesis and characterization of hydrogels of Alg-MA-PBA/spd

To evaluate the mechanical properties of Alg-MA-PBA/Spd hydrogels and Alg-PBA/Spd hydrogels, we used frequency sweep to test their G′ and G″ and the stiffness was compared at 1 Hz (Supplementary Fig. S6A). The results showed that there is no significant difference in their stiffness at 1 Hz (Supplementary Fig. S6B). To suggest the viscoelastic differences between Alg-MA-PBA/Spd hydrogels and Alg-PBA/Spd hydrogels, we measured the stress relaxation of the two hydrogels (Supplementary Fig. S6C). We found that the stress relaxation of Alg-MA-PBA/Spd hydrogels is significantly slower than that of Alg-PBA/Spd hydrogels (Supplementary Fig. S6D). The half stress relaxation time is 1385 s for Alg-MA-PBA/Spd hydrogel, and 38 s for Alg-PBA/Spd hydrogel. We chose Alg-MA-PBA/Spd hydrogel as the elastic control group for viscoelastic Alg-PBA/Spd hydrogel.

### Measurement and simulation of viscoelasticity of PDL *in vivo*

To evaluate the similarity of viscoelasticity between our hydrogels and native PDL tissues, we got a fresh pig tooth-periodontal membrane–bone complex and measured the viscoelasticity of the PDL tissues. The stress relaxation curve of Alg-PBA/Spd hydrogel and PDL tissue has a similar trend, while the stress relaxation curve of Alg-MA-PBA/Spd hydrogel and PDL tissue is quite different (Supplementary Fig. S7A). We further compared their half stress relaxation time, and observed there is no significant difference between Alg-PBA/Spd hydrogel and PDL tissue, but a significant difference between Alg-MA-PBA/Spd hydrogel and PDL tissue (Supplementary Fig. S7B). Alg-PBA/Spd hydrogel can completely cover the stress relaxation range of native PDL tissue. In addition, Alg-MA-PBA/Spd hydrogel can be used as an elastic control group.

### Evaluation of *in vivo* therapeutic effect of Alg-PBA/spd hydrogel in animal models of periodontal defects

**Figure 5.**
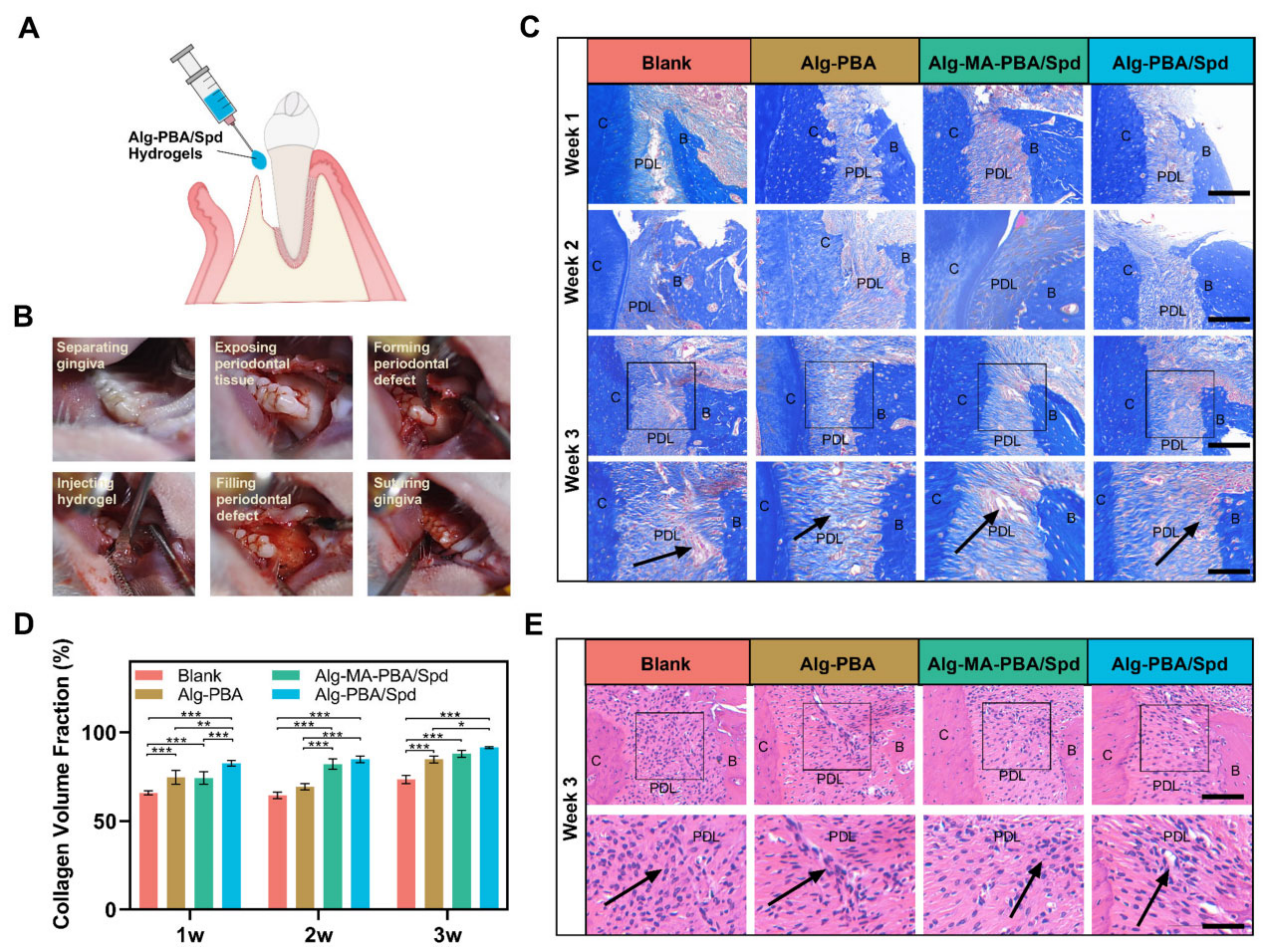
Alg-PBA/Spd hydrogel promotes PDL tissue regeneration *in vivo*. (**A**) Schematic diagram of periodontal defect modeling in rats. (**B**) The mucoperiosteal flap was elevated to expose the alveolar bone on the lingual side of the first maxillary molar. The alveolar bone covering the root surface is removed, creating a periodontal window defect. The Alg-PBA/spd hydrogel is injected at the defect site. After the hydrogel completely filled the defect, the gums were sutured. (**C**) Masson staining images of the periodontal tissues at specific time points (Weeks 1, 2, 3) after different treatments. Scale bar = 200 and 50 μm. PDL, periodontal ligament; B, alveolar bone; C, cementum. (D) The collagen volume fraction of the Masson staining images (a). *P < 0.05, **P < 0.01, ***P < 0.001. (**E**) H&E staining images of the periodontal tissues at specific time points (Week 3) after different treatments. Scale bar = 100 and 50 μm.

To evaluate *in vivo* therapeutic effect of Alg-PBA/Spd hydrogel, we established the periodontal defect with a width of 1.5 mm and a depth of 2 mm was formed around the maxillary first molar of the rat, and the defect was completely filled with hydrogel (Fig. 5A and B). To assess the histology and regeneration of PDL in each group, we performed Masson trichrome staining and hematoxylin and eosin (H&E) staining. The collagen fibers in the PDL space of the four groups show an increasing trend with the prolongation of tissue recovery time (Fig. 5C). In the first week, the total amount of PDL and gingival tissue in the groups of Alg-PBA and Alg-PBA/Spd hydrogel showed greater than those in the groups of blank and Alg-MA-PBA/Spd hydrogel. There were a large number of red-stained muscle fibers in the PDL space of the blank group, but only a small amount of collagen fibers arranged in bundles in the Alg-PBA/Spd group and no similar neatly arranged fiber structures in the other three groups. At the second week, the PDL spaces of all four groups were all filled with new periodontal collagen fibers. The fibers of the Alg-PBA and Alg-PBA/Spd groups arranged regularly with dense fiber bundles, while the fibers in the blank and Alg-MA-PBA/Spd group showed irregularly arranged, even with a lot of muscle fibers in the blank group. At the third week, the proportion of collagen fibers in all four groups increased. The enlarged image in the blank group showed that there were irregular muscle fibers in the local area, with the collagen fibers in the remaining three groups being arranged in a neat fiber bundle structure.

Next, we quantified the volume fraction of collagen and the amount of collagen fibers generated (Fig. 5D). In the early stage of tissue recovery, the volume of collagen in the experimental groups treated with hydrogel showed higher than that in the blank control group, with the highest volume fraction in the Alg-PBA/Spd group. In the middle and late stages of tissue recovery, there was no significant difference in the volume of collagen produced between the Alg-PBA/Spd and Alg-MA-PBA/Spd groups, with those of both groups being significantly higher than the Alg-PBA and blank groups.

Further, we assessed the arrangement of PDL cells by H&E staining (Fig. 5E). As indicated by the arrows, the cells in the blank group were arranged in disorder with clear alveolar bone tissue resorption. The cells in Alg-PBA group were mostly concentrated near the alveolar bone, with dense and regularly arranged fibrous structures. The cells in Alg-MA-PBA/Spd and Alg-PBA/Spd groups were evenly distributed along the fibers, and the fibers were arranged neatly.

Based on the analysis of the direction distribution of collagen fibers in the HE staining results (Supplementary Fig. S5), we found that the direction of fiber distribution in the viscoelastic group (Alg-PBA and Alg-PBA/Spd) is concentrated in the horizontal direction and arranged orderly. The fibers of the elastic group and the blank group (Alg-MA-PBA/Spd and Blank) are distributed in the vertical direction, and the distribution of the elastic group in all directions is relatively small.

We believed that the viscoelastic group can match the viscoelasticity of native PDL. In the process of PDL fiber regeneration, similar viscoelasticity causes the hydrogel to have a smaller external force on the PDL fibers, which helps the fibers to be arranged more orderly. The elastic group hinders the regeneration of PDL fibers, resulting in the disordered arrangement of regenerative PDL fibers. The PDL fibers of the blank group are not affected by external forces and can regenerate in a vertical direction. These results demonstrated that viscoelastic hydrogel might facilitate the generation of PDL fibers in the early stage of repair, and the synergistical treatment with spermidine could even promote the production of collagen by PDLFs and maximize the therapeutic effect.

## Conclusion

In this study, we constructed a double cross-linked hydrogel (Alg-PBA/Spd hydrogel) for PDL repair and regeneration based on the formation of borate ester bonds and B-N ligand bonds. We demonstrated that the mechanical viscoelasticity of Alg-PBA/Spd hydrogel could synergize with biochemical factors (spermidine) to achieve optimal PDL repair. These results highlight the important role of viscoelastic implants in tissue repair, especially in PDL, a tissue subjected to dynamic mechanical stimulation. Based on this work, the coupling between dynamic periodontal stresses and the dynamic viscoelasticity of hydrogels can be explored in the future, which may provide more interesting insights into the mechanisms of PDL repairing using viscoelastic materials. Besides, the role of mechano-biochemically synergistic treatment for tissue regeneration and functional recovery has attracted increasing attention and has been applied in a variety of scenarios (e.g. myocardial infarction, myasthenia gravis, traumatic brain injury). The present study suggests mechanical factors as important design parameters and functional indicators in the field of regenerative medicine. On the other hand, drug (spermidine) loading through dynamic bonding could be insightful, which represents a simple, inexpensive, but effective approach to the sustained release of drugs and integration with hydrogel viscoelasticity. These could inspire more material designs that combine dynamic crosslinking chemistry with other factors (e.g. drug loading, material degradation, cell culture) to create biomaterials with emerging properties for regenerative medicine.

## Supplementary Material

rbad009_Supplementary_Data
