# Supplementary material for: A viscoelastic alginate-based hydrogel network coordinated with spermidine for periodontal ligament regeneration
Source: Regen Biomater. 2023 Feb 14;10:rbad009. doi: 10.1093/rb/rbad009 (PMC10010660; doi:10.1093/rb/rbad009)
Supplement: rbad009_Supplementary_Data [file rbad009_supplementary_data.docx]

**Supplementary information**

**
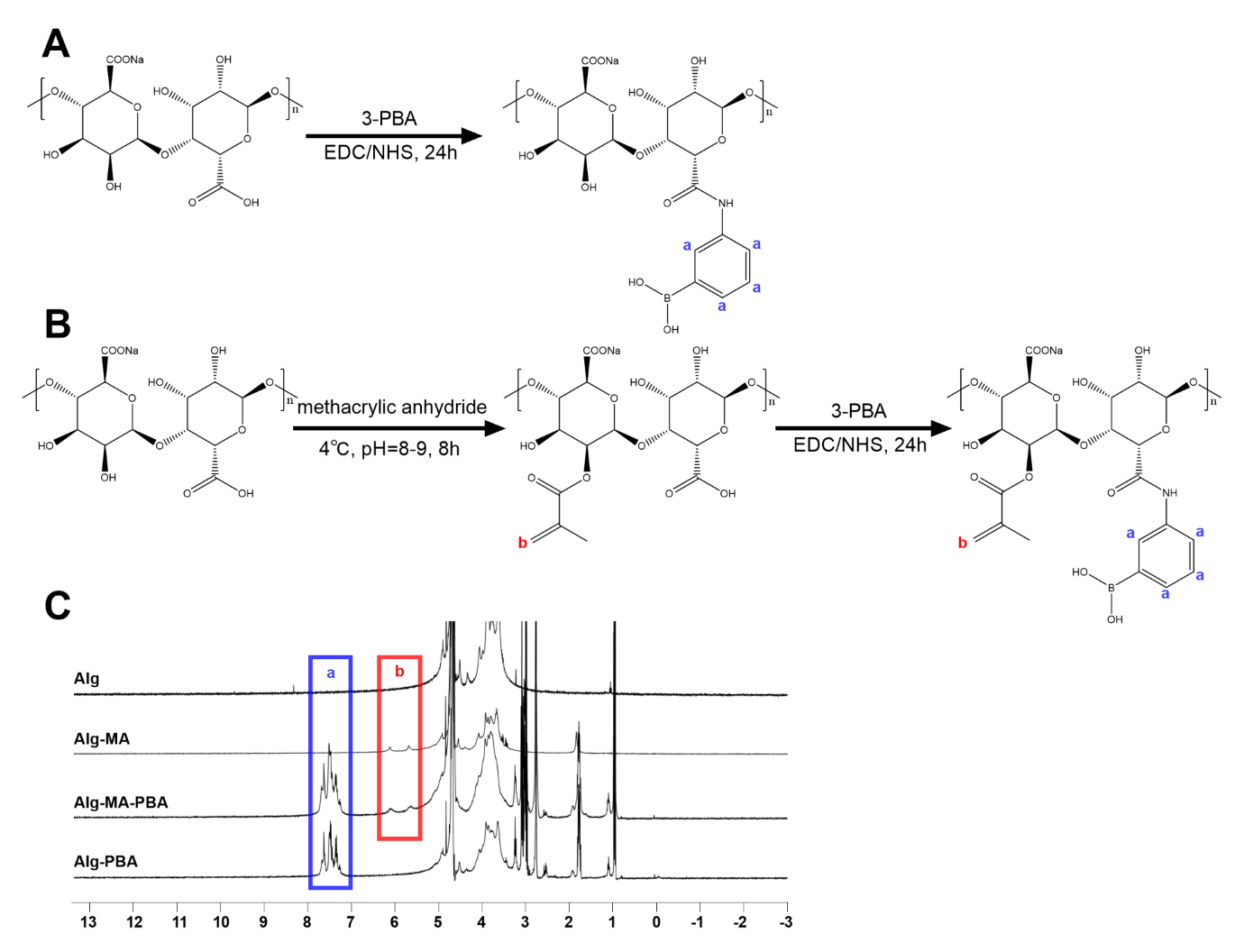
**

**Figure S1. Synthesis and 1H NMR of Alg-PBA and Alg-MA-PBA.** (A-B) Synthesis of Alg-PBA and Alg-MA-PBA. (C) 1H NMR spectra of Alg, Alg-MA, Alg-MA-PBA, Alg-PBA

**
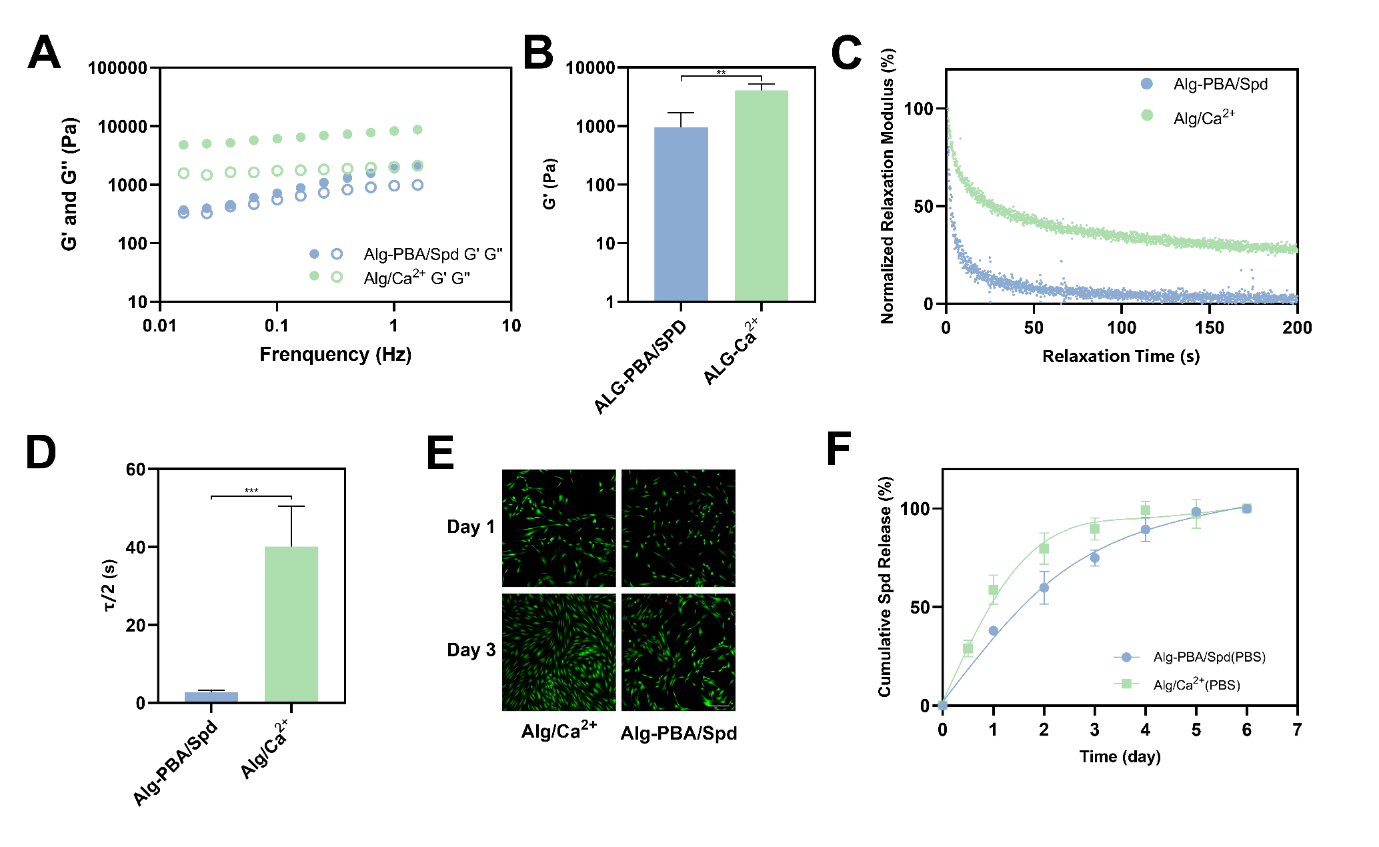
**

**Figure S2. The mechanical features, in vitro biocompatibility and drug release profile between Alg-PBA/Spd and Alg/Ca^2+^.** (A) The frequency sweep of Alg-PBA/Spd and Alg/Ca^2+^. (B) The storage modulus of Alg-PBA/Spd and Alg/Ca^2+^ at 1Hz. (C) The normalized stress relaxation of Alg-PBA/Spd and Alg/Ca^2+^ at 10% strain. (D) The half-stress relaxation time of Alg-PBA/Spd and Alg/Ca^2+^. (E) The Calcein-AM/PI staining of Alg-PBA/Spd and Alg/Ca^2+^ at 1d and 3d. (F) The Spd release profile of Alg-PBA/Spd and Alg/Ca^2+^. All data are shown as mean ± s.d. and compared by unpaired T-test. *, **, ***, **** and ns indicate P < 0.05, P < 0.01, P < 0.001, P < 0.001 and no significant differences, respectively.


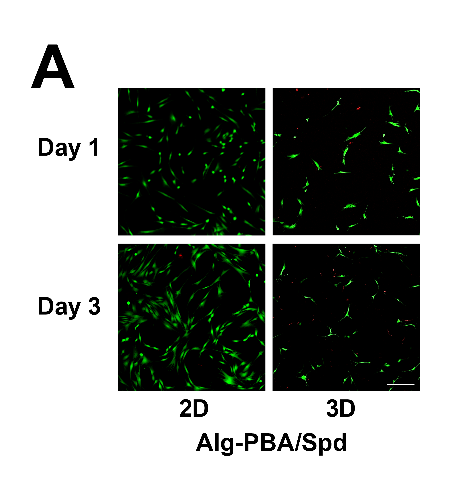


**Figure S3. The biocompatibility of Alg-PBA/Spd.** (A) Live/Dead staining of PDLFs in 2D and 3D culture. Scale bar = 300 μm.


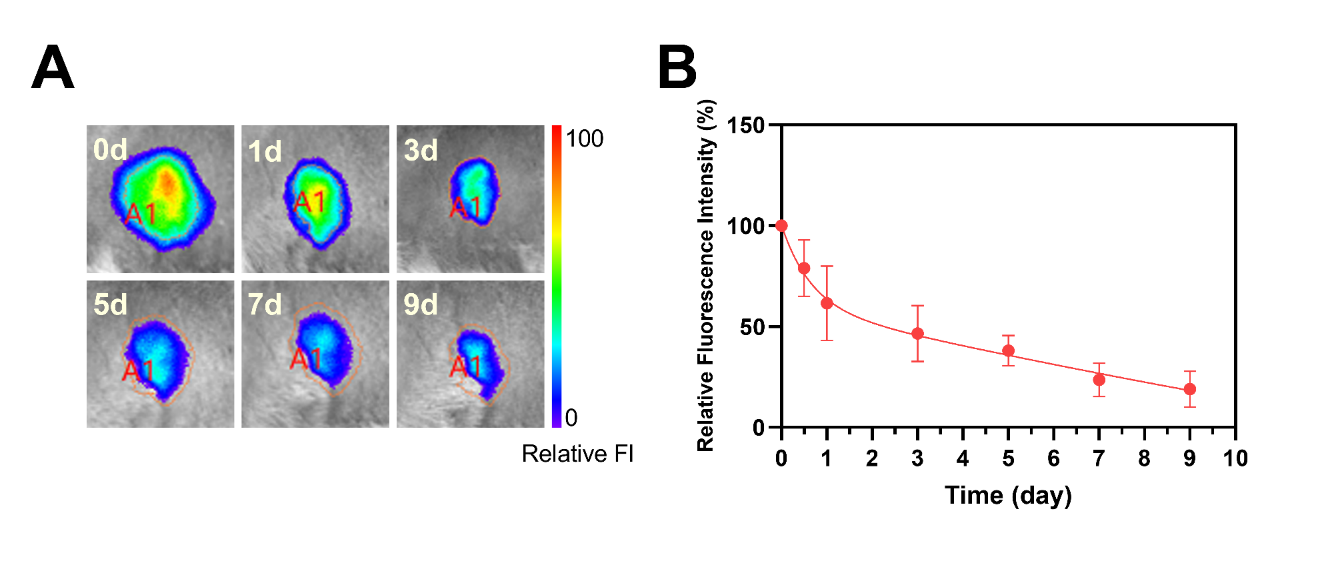


**Figure S4. The degradation of the Alg-PBA/Spd hydrogel in vivo.** (A) Representative relative fluorescence image of hydrogel Cy7-Alg-PBA/Spd hydrogel. (B) Relative fluorescence identify of Cy7-Alg-PBA/Spd hydrogel.

**
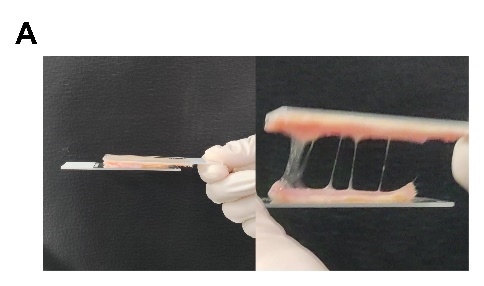
**

**Figure S5. The mucoadhesive features of Alg-PBA/Spd hydrogel.** (A) The photograph of Alg-PBA/Spd hydrogel connecting and separating to the two pieces of skin.


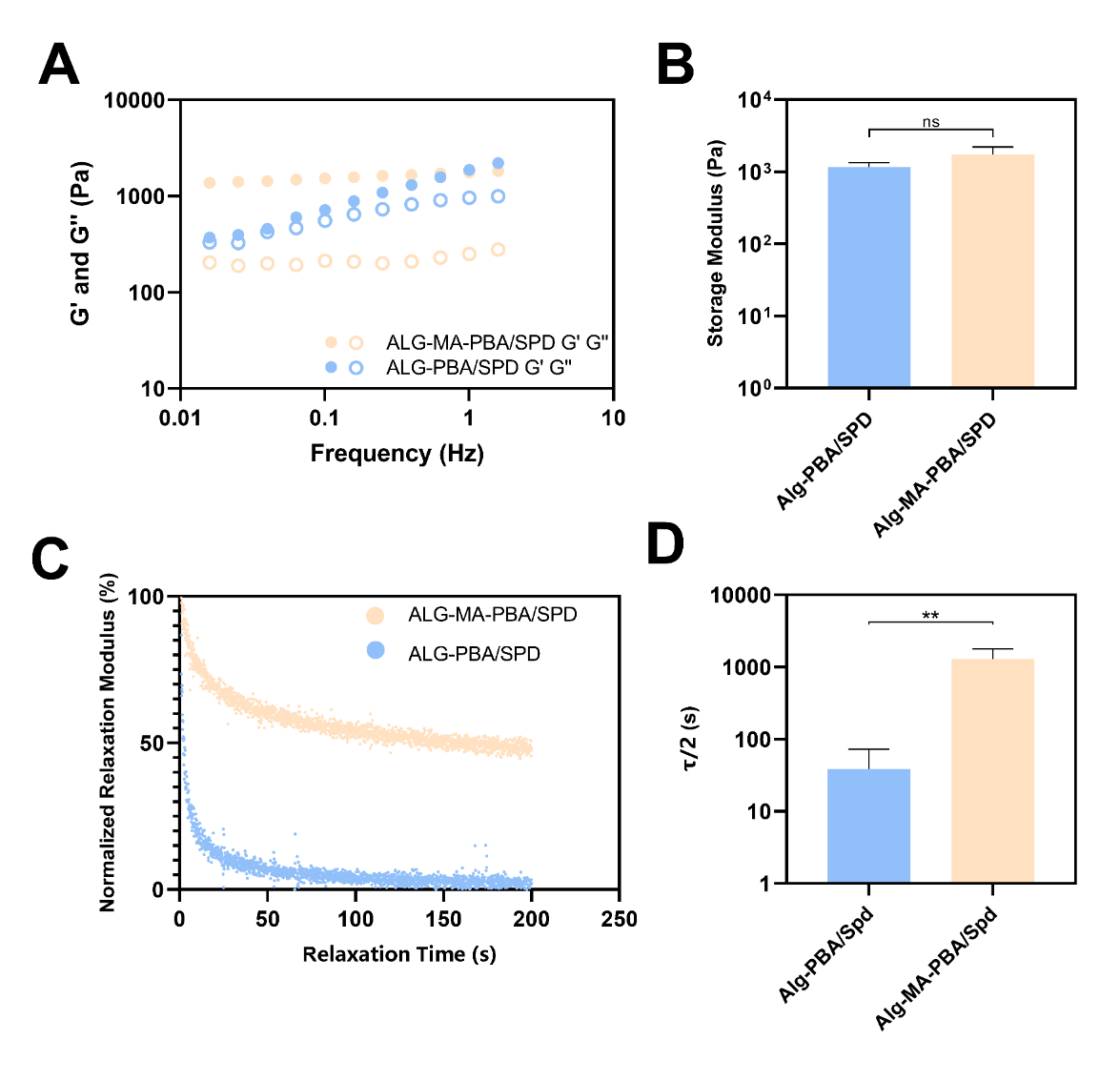


**Figure S6. Comparison of** **mechanical properties between Alg-MA-PBA and Alg-PBA.** (A) The frequency sweep of Alg-PBA/Spd and Alg-MA-PBA from 10Hz to 0.01Hz with 1% strain. (B) The storage modulus of Alg-PBA/Spd and Alg-MA-PBA at 1Hz. (C) The normalized relaxation modulus of Alg-PBA/Spd and Alg-MA-PBA with 1% strain for 200s. (D) The half stress relaxation time of Alg-PBA/Spd and Alg-MA-PBA. Data are shown as mean ± s.d. and compared using a two-tailed Student’s t-test. ns, ** indicates P > 0.05, <0.01, respectively.


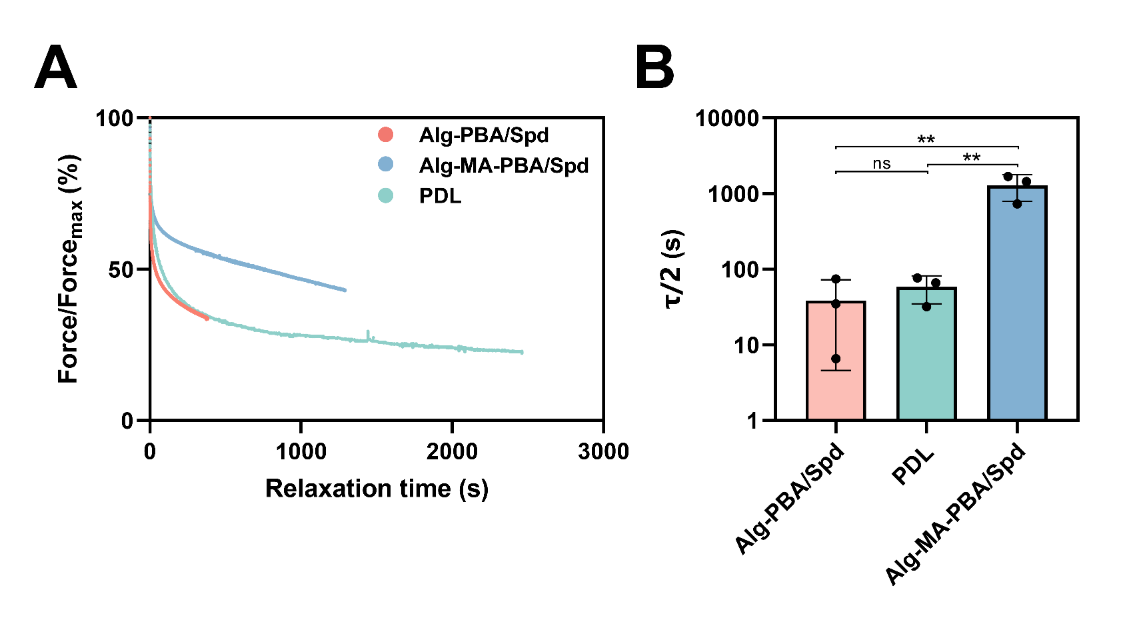


**Figure S7. Measurement and simulation of viscoelasticity of PDL.** (A) Stress relaxation curves of Alg-PBA/Spd, Alg-MA-PBA/Spd and PDL among 3000s. (B) Comparisons of half stress relaxation time of Alg-PBA/Spd, Alg-MA-PBA/Spd and PDL. Data are shown as mean ± SD. and compared using one-way ANOVA followed by Bonferroni’s post hoc test. ns, *, ** and *** indicate P > 0.05, P <0.05, P <0.01 and P <0.001, respectively.

**
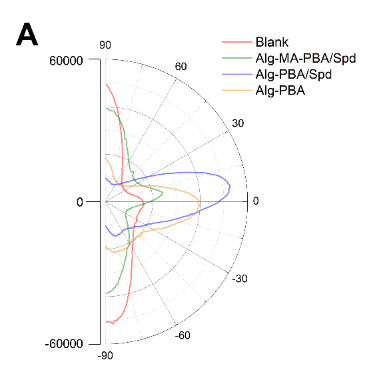
**

**Figure S8. The orientation of PDL fibers distribution.** (A) The orientation of PDL fibers distribution in group Blank, Alg-MA-PBA/Spd, Alg-PBA/Spd and Alg-PBA.
